# Supplementary material for: Time-Restricted Eating Without Exercise Enhances Anaerobic Power and Reduces Body Weight: A Randomized Crossover Trial in Untrained Adults
Source: Nutrients. 2025 Sep 20;17(18):3011. doi: 10.3390/nu17183011 (PMC12473138; doi:10.3390/nu17183011)
Supplement: Supplementary file 1 [file nutrients-17-03011-s001.zip › CONSORT_Crossover_Checklist.pdf]

CONSORT 2010 Checklist of Information to Include When Reporting a Randomized Crossover Trial

Item No.	Checklist item	Reported on page No.
Title and abstract		
1a	Identification as a randomized trial in the title	1
1b	Structured summary of trial design, methods, results, and conclusions	1
Introduction		
2a	Scientific background and explanation of rationale	1–2
2b	Specific objectives or hypotheses	2–3
Methods		
3a	Description of trial design (such as parallel, factorial) including allocation ratio	3
3b	Important changes to methods after trial commencement (such as eligibility criteria), with reasons	Not applicable
4a	Eligibility criteria for participants	3–4
4b	Settings and locations where the data were collected	3–4
5	The interventions for each group with sufficient details to allow replication	3
6a	Completely defined pre-specified primary and secondary outcome measures	4–5
6b	Any changes to trial outcomes after the trial commenced, with reasons	Not applicable
7a	How sample size was determined	3
7b	When applicable, explanation of any interim analyses and stopping guidelines	Not applicable
8a	Method used to generate the random allocation	3

	sequence	
8b	Type of randomisation; details of any restriction	3
9	Mechanism used to implement the random allocation sequence	3
10	Who generated the allocation sequence, enrolled participants, and assigned them to interventions	3
11a	Blinding after assignment to interventions (who was blinded and how)	3
11b	If relevant, description of the similarity of interventions	Not applicable
12a	Statistical methods used to compare groups for primary and secondary outcomes	5
12b	Methods for additional analyses, such as subgroup analyses and adjusted analyses	5
Results		
13a	For each group, the numbers of participants who were randomly assigned, received intended treatment, and were analysed	3
13b	For each group, losses and exclusions after randomisation, with reasons	5
14a	Dates defining the periods of recruitment and follow-up	3
14b	Why the trial ended or was stopped	Not applicable
15	A table showing baseline demographic and clinical characteristics for each group	5
16	For each group, number of participants included in each analysis and whether the analysis was by original	3

	assigned groups	
17a	For each primary and secondary outcome, results for each group, and the estimated effect size and its precision	5
17b	For binary outcomes, presentation of both absolute and relative effect sizes	Not applicable
18	Results of any other analyses performed, including subgroup and adjusted analyses	6–8
19	All important harms or unintended effects in each group	Not applicable
Discussion		
20	Trial limitations, addressing sources of potential bias and imprecision	11
21	Generalisability of the trial findings	11
22	Interpretation consistent with results, balancing benefits and harms	10–11
Other information		
23	Registration number and name of trial registry	UMIN000059063；UMIN Clinical Trials Registry, UMIN-CTR
24	Where the full trial protocol can be accessed	3-4
25	Sources of funding and other support; role of funders	This study received no external funding.
