# Supplementary figures and images for: Time-Restricted Eating Without Exercise Enhances Anaerobic Power and Reduces Body Weight: A Randomized Crossover Trial in Untrained Adults
Source: Nutrients. 2025 Sep 20;17(18):3011. doi: 10.3390/nu17183011 (PMC12473138; doi:10.3390/nu17183011)

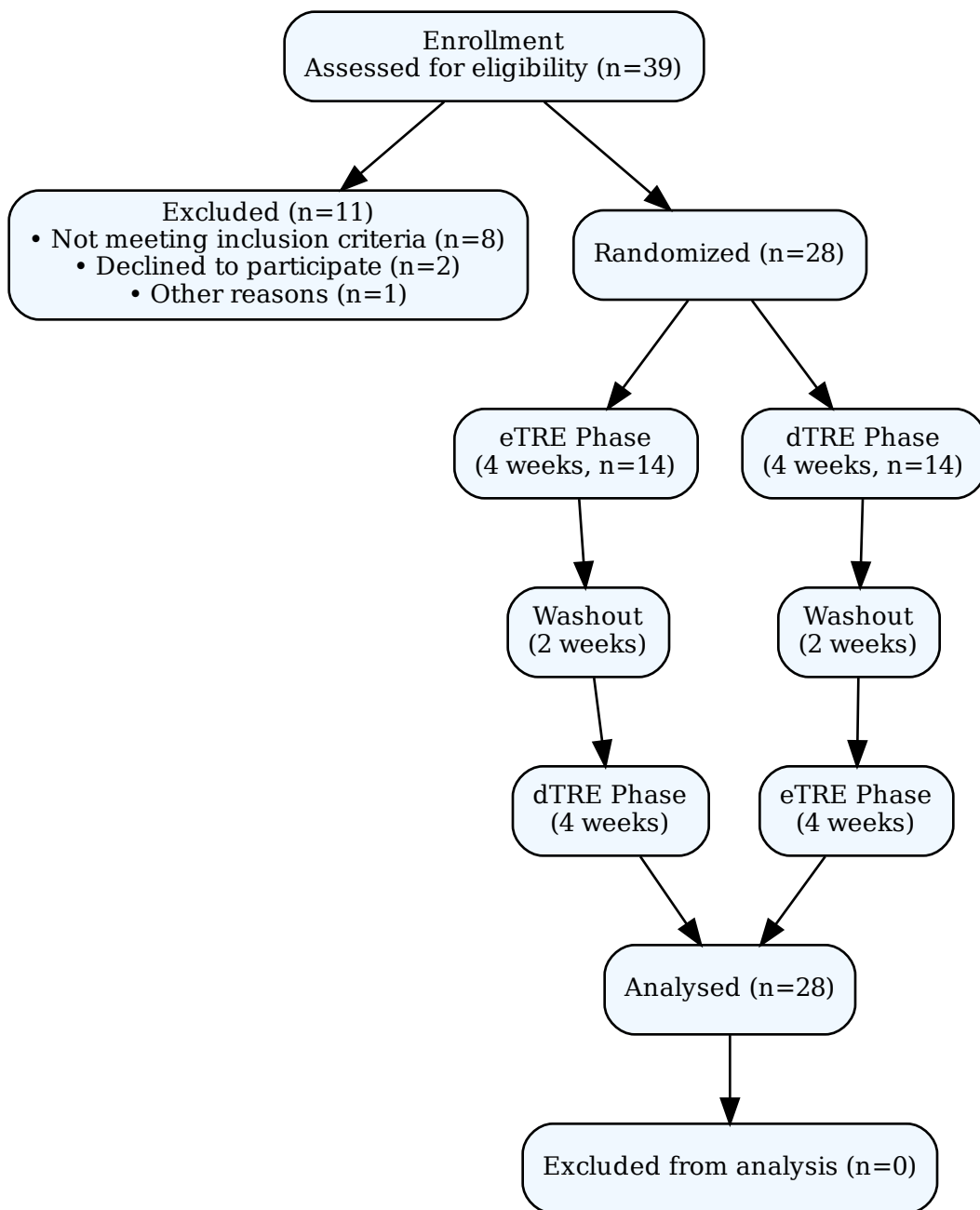

Supplement: Supplementary file 1 [file nutrients-17-03011-s001.zip › consort_flow-diagram.pdf]
